# Deubiquitinase OTUD5 modulates mTORC1 signaling to promote bladder cancer progression

**DOI:** 10.1038/s41419-022-05128-6

**Published:** 2022-09-09

**Authors:** Tao Hou, Weichao Dan, Tianjie Liu, Bo Liu, Yi Wei, Chenyang Yue, Taotao Que, Bohan Ma, Yuzeshi Lei, Zixi Wang, Jin Zeng, Yizeng Fan, Lei Li

**Affiliations:** 1 Department of Urology, the First Affiliated Hospital of Xi’an Jiaotong University, Xi’an, Shaanxi 710061 P.R. China; 2 Xi’an Jiaotong university, Key Laboratory of Environment and Genes Related to Diseases, Ministry of Education, Xi’an, Shaanxi China; 3 The First Affiliated Hospital of Xi’an Jiaotong University, Key Laboratory of Environment and Genes Related to Diseases, Ministry of Education, Xi’an, Shaanxi China; 4 Department of Biology, York University, Toronto, ON M3J1P3 Canada

**Keywords:** Bladder cancer, Ubiquitylation

## Abstract

The mechanistic (formally “mammalian”) target of rapamycin (mTOR) pathway serves as a crucial regulator of various biological processes such as cell growth and cancer progression. In bladder cancer, recent discoveries showing the cancer-promoting role of mTOR complex 1 have attracted wide attention. However, the regulation of mTOR signaling in bladder cancer is complicated and the underlying mechanism remains elusive. Here, we report that the deubiquitinating enzyme, ovarian tumor domain-containing protein 5 (OTUD5), can activate the mTOR signaling pathway, promote cancer progression, and show its oncogenic potential in bladder cancer. In our study, we found that OTUD5 deubiquitinated a RING-type E3 ligase, RNF186, and stabilized its function. In addition, the stabilization of RNF186 further led to the degradation of sestrin2, which is an inhibitor of the mTOR signaling pathway. Together, we provide novel insights into the pathogenesis of bladder cancer and first prove that OTUD5 can promote bladder cancer progression through the OTUD5-RNF186-sestrin2-mTOR axis, which may be exploited in the future for the diagnosis and treatment of this malignancy.

## Introduction

Bladder cancer places a substantial burden on society with nearly 170,000 deaths per year worldwide [1]. Although the risk factors of its occurrence remain to be elucidated, recent development in sequencing technology has identified several genes and pathways which are key drivers of bladder cancer, including cell cycle-related genes, RAS, and PI-3-kinase/mTOR [2].

The mechanistic target of rapamycin (mTOR) signaling pathway plays an important role in homeostasis and coordinates intracellular and extracellular signals to control cell metabolism, growth, and proliferation [3, 4]. Specifically, mTOR complex 1 (mTORC1) activation is triggered by several upstream signals such as growth factors, energy, and nutrients. Activated mTORC1 can then phosphorylate a variety of downstream targets, including S6K, 4EBP1, TFEB, and ULK1, thereby regulating cell growth, metabolism and autophagy [5]. Not surprisingly, dysregulated mTORC1 signaling is closely related to various diseases including cancers, metabolic diseases and developmental disorders [6].

Recently, post-translational modifications such as ubiquitination and deubiquitination of mTOR signaling components have emerged as a promising research area in mTOR signaling regulation [7]. For example, the tumor suppressor SCF^Fbw7^ ubiquitin ligase has been observed to target mTOR for ubiquitination and degradation [8]. In addition, DEPTOR as an inhibitor of mTORC1 and mTORC2 can be the substrate of E3 ligase SCF^βTrCP^, triggering DEPTOR ubiquitination and degradation [9–11]. Recently, some studies have found that deubiquitinating enzymes (DUBs) can remove the ubiquitin chains on mTOR-interacting proteins and regulate mTOR signaling. The above-mentioned DEPTOR can be deubiquitinated and stabilized by ovarian tumor domain-containing ubiquitin aldehyde-binding protein 1 (OTUB1) belonging to the OTU family DUBs, leading to the inhibition of mTOR signaling [12]. Furthermore, ubiquitin-specific peptidase 9, X-linked (USP9X) can negatively regulate mTOR signaling through the interaction with mTOR, RAPTOR and RICTOR [13].

The OTU family DUBs have been the focus in many studies and shown to function in numerous cellular processes. Recent progress in elucidating the functions of OTUD5 as a member of this family has greatly enhanced our understanding in this area. The first discovered function of OTUD5 is to cleave the polyubiquitin chains on TRAF3 to negatively regulate IFN-I expression [14]. In addition, OTUD5 can promote DNA damage repair by stabilizing KU80 [15], as well as regulate DNA damage response by regulating FACT-dependent transcription on damaged chromatin [16]. Meanwhile, OTUD5 can enhance anti-tumor and anti-viral immunity by deubiquitinating and stabilizing STING [17]. However, there are few reports addressing the function of OTUD5 in tumorigenesis, especially in bladder cancer.

In this study, we proved that OTUD5 is involved in the progression of bladder cancer and exerts its cancer-promoting effect by activating mTORC1 signaling. Additionally, our research revealed the OTUD5-RNF186-sestrin2-mTOR axis and provided novel insights into the diagnosis and treatment of bladder cancer.

## Materials and Methods

### Cell culture

Human bladder cancer cell lines 253 J, UM-UC-14 and T24 were purchased from American Type Culture Collection (ATCC; Manassas, VA, USA) and cultured in Dulbecco’s modified Eagle’s medium (DMEM) (Gibco; Thermo Fisher Scientific, Inc.) supplemented with 10% fetal bovine serum (FBS) (Gibco; Thermo Fisher Scientific, Inc.). The HEK293T cell line was obtained from Professor Chawnshang Chang (Department of Urology, University of Rochester, Rochester, NY 14642, USA) and maintained in DMEM supplemented with 10% FBS. In addition, the culture medium was supplemented with 100 U/mL penicillin and 0.1 mg/ml streptomycin (Gibco; Thermo Fisher Scientific, Inc.).

### Transfection and establishment of stable clone cells

T24 cells were stably transfected with *OTUD5* (T24-OTUD5) and 253 J, UM-UC-14 cells were stably transfected with sh-*OTUD5* (253J-shOTUD5, UM-UC-14-shOTUD5), and empty vector control sublines (T24-Vec, 253J-shNC, UM-UC-14-shNC) were established. Cells were transfected with various plasmids using Lipofectamine 2000 transfection reagent (Life Technologies, 11668-027) according to the manufacturer’s protocol. Various cell lines were infected with lentiviral and retroviral cDNA expressing viruses, which were packaged in HEK293T cells. Then, hygromycin B (200 μg/ml) or puromycin was used to select infected cells (1 μg/ml). All established cell lines were cultured for less than 6 months and tested for mycoplasma every month.

### Reagents, antibodies and plasmids

Everolimus (SML2282) was purchased from Sigma-Aldrich; Merck KGaA and dissolved in DMSO. Dynabeads Protein G (10004D) and TRIzol reagent were purchased from Invitrogen. Antibodies against OTUD5 (PA5-20611) and RNF186 (PA5-42330, PA5-57315) were purchased from Thermo Fisher Scientific. Antibodies against mTOR (2983), Phospho-mTOR (Ser2448) (5536,2976), Phospho-p70 S6 Kinase (Thr421/Ser424) (9204), Phospho-4E-BP1 (Thr37/46) (2855), Cleaved-PARP (5625), Cleaved-caspase3 (9664), Flag-tag (14793), HA-tag (3724), GST-tag (2624) and β-actin (3700) were purchased from Cell Signaling Technology. Antibodies against Ki-67 (ab92742) and Bax (ab32503) were purchased from Abcam. Antibody against sestrin2 (10795-1-AP) was purchased from Proteintech Group. *pcDNA3-Flag-OTUD5*, *pcDNA3-HA-SESN1*, *pcDNA3-HA-SESN2*, *pcDNA3-HA-SESN3*, *pcDNA3-HA-RNF186* and *pGEX-4T-1-GST-OTUD5* plasmids were constructed according to our standard protocols. The construct expressing His-ubiquitin was purchased from Addgene. *pLKO-*sh*OTUD5* (TRCN0000233196, TRCN0000143838) was purchased from Sigma-Aldrich.

### Total RNA extraction and quantitative real-time PCR

Total RNA of bladder cancer cells was extracted with TRIzol. 2 μl of extracted RNA was used for RNA quantification and was reverse transcribed using a Reverse Transcription Reaction Kit (TaKaRa PrimeScriptTM RT Master Mix). Then cDNA was amplified using specific primers. Primer sequences are listed as follows:

*OTUD5* (forward primer, 5ʹ- GGTTGTGCGAAAGCATTGCAT-3ʹ; reverse primer, 5ʹ- ACCTCCACAGGACGGTTGT-3ʹ) and *ACTB/actin beta* (forward primer, 5ʹ-CATGTACGTTGCTATCCAGGC-3ʹ; reverse primer, 5ʹ-CTCCTTAATGTCACGCACGAT-3ʹ). Using *ACTB* as an internal reference, relative changes in gene expression were normalized against *ACTB*.

### Western blot analysis

Cells were washed with ice-cold PBS and lysed with radioimmunoprecipitation assay (RIPA) buffer (50 mM Tris, 150 mM NaCl, 0.1% SDS, 1% NP40 and 0.5% sodium deoxycholate; pH 7.4) containing protease inhibitors (Sigma-Aldrich; Merck KGaA) and phosphatase inhibitors (Sigma-Aldrich; Merck KGaA). For immunoblotting analysis, 20-40 μg samples of protein (total lysate and mitochondriallysosomal, cytoplasmic and nuclear fractions) are subjected to SDS-PAGE on a 10% or 15% Tris-glycine gel. The separated proteins were then transferred to polyvinylidene fluoride (PVDF) membranes by Western blotting. The membrane was blocked with 5% milk at room temperature for 1 hour, and then incubated with a primary antibody at 4 °C overnight, and then incubated with a peroxidase-conjugated secondary antibody at room temperature (25 °C) for 1 hour. Finally, the immune response signal of the protein was detected by the ECL detection system (Thermo Fisher Scientific, Rochester, NY).

### Confocal fluorescence microscopy and Immunofluorescence staining

The cells were planted on glass slides and transiently transfected with *Flag-OTUD5*, *HA-SESN2* and *HA-RNF186* plasmids for 48 h. After washing three times with pre-cold phosphate buffered saline (PBS), the cells were treated with 4% paraformaldehyde for 15 min. The cells were then permeabilized with 0.1% Triton X-100 and incubated with specific primary antibodies targeting OTUD5, sestrin2 and RNF186 at a dilution of 1:200 overnight at 4 °C. At room temperature, the cells were stained with fluorescein isothiocyanate (FITC)- and tetramethyl rhodamine isothiocyanate (TRITC)-conjugated secondary antibodies for 1 h. Then, the cells were stained with DAPI and blocked with glycerol. The fluorescence microscope was used to detect the fluorescence of the cells.

### MTT assay

Bladder cancer cells were plated into 96-well culture plates at the cell density of 5.0 × 10^4^/ml. After 6 h, 24 h, 48 h, 72 h, and 96 h, the supernatant was changed with fresh medium containing 10% MTT (5 mg/ml) for another 4 h incubation. Then, the supernatant was removed and 150 µL DMSO was added into each well. The 96-well microplate reader (Bio-Rad, Hercules, USA) was used to detect the absorbance at the wavelength of 490 nm.

### Colony formation assay

Bladder cancer cells in the logarithmic growth phase were seeded into each well of a 6-well plate (1,000 cells/well) and cultured with DMEM medium supplemented with 10% FBS in an incubator at 37 °C with 5% CO_2_. 1 week after seeding when colony formation became visible, the medium was discarded and the colonies were washed thrice with PBS, then fixed with 4% paraformaldehyde for 15 min and stained with crystal violet for 15 min. Then, the staining solution was slowly washed away with running water. After the plate was air dried, the number of colonies was determined.

### Enrichment analysis

We used Metascape (http://metascape.org/) online tool for pathway enrichment of the proteins analysed by mass spectrometry profiling, and the pathway gene sets used for enrichment contained Canonical Pathways, Hallmark Gene Sets, Reactome Gene Sets, KEGG Pathway, WikiPathways, with parameters Min Overlap = 3, *P* Value Cutoff = 0.01, Min Enrichment = 1.5.

### Flow cytometry analysis

We used FITC Annexin V Apoptosis Detection Kit I to measure cell apoptosis and performed the assay according to the manufacturer’s instructions. Meanwhile, the apoptotic cells were analysed by flow cytometry (BD FACScan flow cytometer, BD Biosciences).

### Ethynyl deoxyuridine incorporation assay

Cell proliferation was measured using the BeyoClick™ EdU Cell Proliferation Kit with Alexa Fluor 488 (C0071S, Beyotime, China) according to the manufacturer’s instructions. The fluorescence microscope was used to analyse the results. The EdU-positive cells were counted in at least four randomly selected fields.

### Protein structure prediction and protein-protein docking

The full-length structures of OTUD5 and RNF186 were predicted by trRosetta. The interface was visualised using PyMOL, and the detailed analysis of the OTUD5/RNF186 complex was conducted using PDBePISA. ΔG (kcal/mol) indicates the solvation free energy gain upon formation of the interface. ΔiG *P* value indicates the *P* value of the observed solvation free energy gain. The *P* value measures the probability of getting a value lower than the observed ΔiG, when the interface atoms are picked randomly from the protein surface, such as to amount to the observed interface area.

### Co-immunoprecipitation

After being transfected with the specific plasmid, the cells were lysed with IP buffer [50 mM Tris HCl, 150 mM NaCl, 1 mM ethylenediaminetetraacetic acid (EDTA), 1% Triton X-100] containing protease inhibitors (Sigma-Aldrich; Merck KGaA) and phosphatase inhibitors (Sigma-Aldrich; Merck KGaA). After incubation with M2 agarose beads (Sigma-Aldrich) bound with anti-FLAG or anti-HA antibodies for 4 h at 4 °C with gentle shaking, the cell lysate was washed with IP buffer, and the protein was extracted from the beads by boiling at 95 °C for 5 min.

### In vivo ubiquitination assays

293 T cells were transfected with plasmid encoding His-tagged ubiquitin. 42 hours after transfection, cells were treated with 20 mM MG132 for 6 hours. Then, cells were lysed with buffer A [6 M guanidine-HCl, 0.1 M Na_2_HPO4/NaH_2_PO4, and 10 mM imidazole (pH 8.0)] and sonicated for 15 seconds. After incubating with nickel-nitrilotriacetic acid (Ni-NTA) beads (QIAGEN) for 3 hours at room temperature, the protein was washed twice with buffer A, twice with buffer A/TI (1 volume of buffer A mixed with three volumes of buffer TI), and then eluted once with buffer TI [25 mM Tris-HCl and 20 mM imidazole (pH 6.8)]. The pulled-down protein was denatured by boiling at 95 °C for 5 min and immunoblotted following SDS-PAGE separation.

### Bladder cancer xenograft animal model

All animal experiments were approved by the Institutional Animal Care and Use Committee of Xi’an Jiaotong University and their care was in accordance with institution guidelines. 4-week-old BALB/c male nude mice were purchased from the Experimental Animal Center of Xi’an Jiaotong University. The cultured UM-UC-14/shNC, UM-UC-14/shOTUD5-01 and UM-UC-14/shOTUD5-02 cells were detached by trypsinization, washed, and resuspended in serum-free DMEM medium containing matrigel (Sigma-Aldrich; Merck KGaA). The cells (2 × 10^6^ cells in 100 μL) were then injected subcutaneously into the right flank of the nude mice to initiate tumor growth. Seven days after inoculation, tumor size was measured by caliper every three days. The mice were separated into two groups: UM-UC-14/shNC, UM-UC-14/shOTUD5-01 and UM-UC-14/shOTUD5-02 control group (*n* = 6); and UM-UC-14/shNC, UM-UC-14/shOTUD5-01 and UM-UC-14/shOTUD5-02 everolimus treatment group (2.5 mg/kg, *n* = 6). All mice were subjected to operation every three days, and the tumor volume was calculated as follows: volume (mm^3^) = 1/2 × (length) × (width)^2^. All treatments were administered for 30 days. At the end of the experiment, tumors were excised, weighed, and then fixed in 4% paraformaldehyde or stored in liquid nitrogen for further analyses.

### Immunohistochemical assay

The 60 BCa specimens were obtained from the First Affiliated Hospital of Xi’an Jiaotong University and the usage of these specimens was approved by the Institute Review Board of the First Affiliated Hospital of Xi’an Jiaotong University. For IHC, in brief, the samples were deparaffinized and incubated with primary antibody against OTUD5 or p-mTOR. After incubating with a horseradish peroxidase-conjugated secondary antibody at room temperature for 1 h, the specimens were stained with diaminobenzidine (DAB) and evaluated under the microscope (Olympus Optical Co., Ltd., Tokyo, Japan).

### Statistical analysis

All data were presented as mean ± standard deviation (SD) of 3 independent experiments. All statistical analyses were performed using GraphPad Prism 5.2 software (GraphPad Software, Inc.). The difference between two groups was analysed by Student’s *t* test. The difference among multiple groups was analysed by one-way analysis of variance. *P* < 0.05 was used to suggest statistical significance.

## Results

### OTUD5 is overexpressed and acts as an oncogene in bladder cancer

**Fig. 1:**
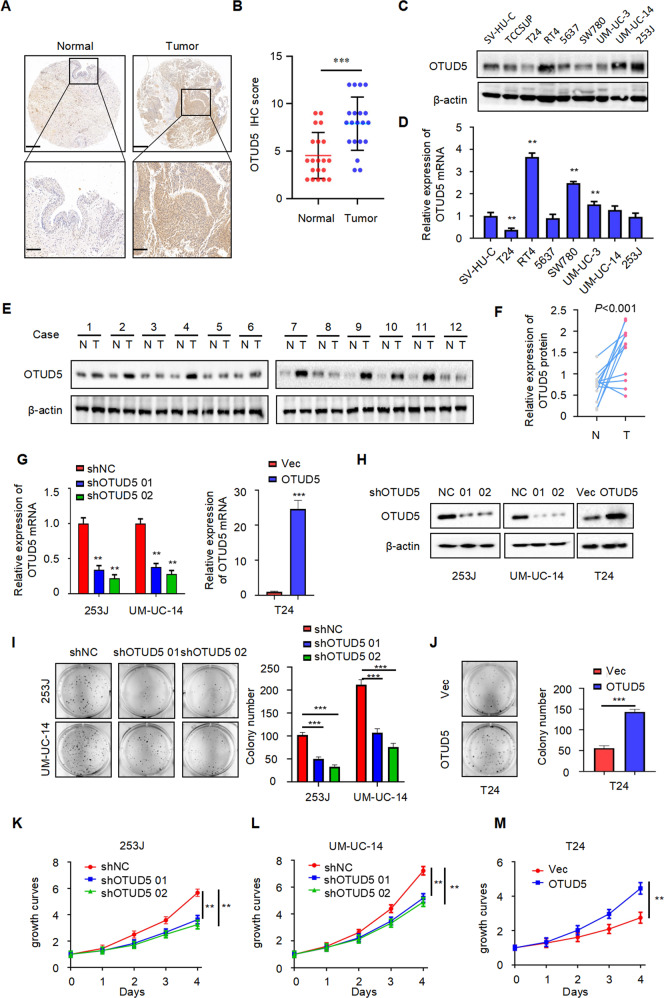
OTUD5 is overexpressed and acts as an oncogene in bladder cancer. **A** Representative images of OTUD5 immunohistochemistry (IHC) staining in bladder cancer tissue and adjacent normal bladder tissue samples. Scale bars, upper 500 μm, lower 100 μm. **B** Statistical analysis of OTUD5 expression in adjacent normal bladder tissues and bladder cancer tissues. ***P < 0.001. **C**, **D** Western blot analysis and quantitative real-time RT-PCR of OTUD5 expression in the normal human bladder cell line and bladder cancer cell lines. **E** Western blot analysis of OTUD5 protein expression in adjacent normal bladder tissues and cancer tissues. β-actin was used as the loading control. **F**. The quantitative analysis of the relative expression of OTUD5 protein. **G**, **H** Quantitative real-time RT-PCR and Western blotting analysis of OTUD5 expression in 253 J or UM-UC-14 cell lines transfected with OTUD5 shRNAs and shNC, and T24 cell line infected with OTUD5 lentivirus and negative control. 18 S was applied as the endogenous control for quantitative real-time RT-PCR, and β-actin was used as a loading control for Western blotting assay. **I** Colony formation assays and quantification results of 253 J and UM-UC-14 cells with OTUD5 knockdown. ***P < 0.001. **J** Colony formation assays and quantification results of T24 cells with OTUD5 overexpression. Vec vector. ***P < 0.001. **K**, **L**, **M** MTT assay was performed to detect viability in 253 J and UM-UC-14 cells with OTUD5 knockdown and T24 cells with OTUD5 overexpression. **P < 0.01. All data are presented as mean ± SD of 3 independent experiments.

To explore the role of OTUD5 in bladder cancer, we first performed immunohistochemical analysis of tissue samples from patients with bladder cancer (Fig. 1A, B). The result showed significantly higher OTUD5 expression in bladder cancer tissues compared with the adjacent tissues. Furthermore, Western blot (Fig. 1C) and RT-PCR (Fig. 1D) analyses showed increased OTUD5 expression in bladder cancer cell lines (RT4, 5637, UM-UC-14 and 253 J, not including T24) compared with the urothelial cell line SV-HUC. Additionally, we analysed the expression of OTUD5 in paired samples of bladder cancer tissues and normal bladder tissues from 12 patients through Western blot. The result once again showed increased OTUD5 expression in bladder cancer tissues (Fig. 1E, F). Next, we investigated the role of OTUD5 in bladder cancer by constructing cell lines with stable OTUD5 knockdown and overexpression (Fig. 1G, H). Plate cloning experiment using 253 J and UM-UC-14 cells showed reduced cell proliferation in the OTUD5 knockdown group when compared with the control group (Fig. 1I). In contrast, plate cloning experiment using T24 cells showed enhanced cell proliferation in the OTUD5 overexpression group when compared with the control group (Fig. 1J). Further MTT assays yielded results similar to those shown in the plate cloning experiment (Fig. 1K–M). These results indicate that *OTUD5* may play a cancer-promoting role in bladder cancer, and it may be involved in the occurrence and development of bladder cancer as an oncogene.

### OTUD5 promotes proliferation in bladder cancer

**Fig. 2:**
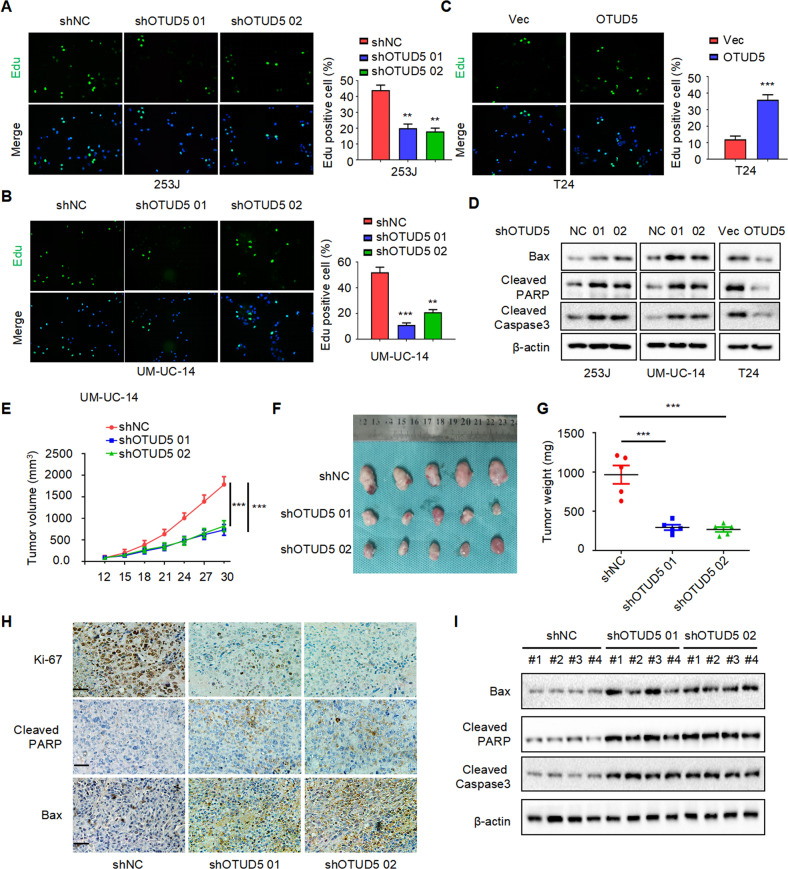
OTUD5 promotes proliferation in bladder cancer. **A**, **B**, **C** EdU assay was performed to detect proliferation in 253 J and UM-UC-14 cells with OTUD5 knockdown and T24 cells with OTUD5 overexpression. **P < 0.01, ***P < 0.001. **D** Western blot was performed to determine the effect of OTUD5 on the expression of apoptosis markers (Bax, Cleaved-PARP, Cleaved-Caspase3) in bladder cancer cells. All data are presented as mean ± SD of three independent experiments. **E** The growth curves of xenografts in different groups. Tumor volumes were measured every three days from day 12 to day 30. ***P < 0.001. **F** Illustration of tumors excised from male nude mice in each group. **G** After 30 days, the nude mice were sacrificed, and UM-UC-14 cell xenografts were weighed. ***P < 0.001. **H** Immunohistochemical analysis of Ki67, Bax and Cleaved-PARP in tumor xenografts. **I** Western blot analysis of Bax, Cleaved-PARP and Cleaved-Caspase3 expression in tumor xenografts.

Furthermore, we investigated the function of OTUD5 in bladder cancer and found that OTUD5 can promote the proliferation of bladder cancer cells. We used the previously constructed cell lines as the research object. Through EdU experiments, we found that knocking down OTUD5 could inhibit proliferation in 253 J and UM-UC-14 cells, and overexpressing OTUD5 could promote the proliferation in T24 cells (Fig. 2A–C). Meanwhile, by analysing apoptosis-related markers through Western blot, we found knocking down OTUD5 can promote the expression of Bax, Cleaved-PARP and Cleaved-caspase3, while overexpressing OTUD5 can inhibit their expression (Fig. 2D). Similarly, we detected apoptotic responses through flow cytometry and found that knocking down OTUD5 can promote apoptosis in 253 J and UM-UC-14 cells, while overexpressing OTUD5 could inhibit apoptosis in T24 cells (Supplementary Fig. 1I–K). As shown in Fig. 2E–G, OTUD5 knockdown can inhibit xenograft growth in vivo. Additionally, immunohistochemistry indicated that OTUD5 knockdown promoted the expression of Ki67, Bax and Cleaved-PARP (Fig. 2H). Upregulation of Bax, Cleaved-PARP and Cleaved-Caspase3 was further confirmed by Western blot (Fig. 2I). Taken together, these results indicate that OTUD5 can promote tumor proliferation in bladder cancer.

### OTUD5 positively regulates the mTOR signaling pathway

**Fig. 3:**
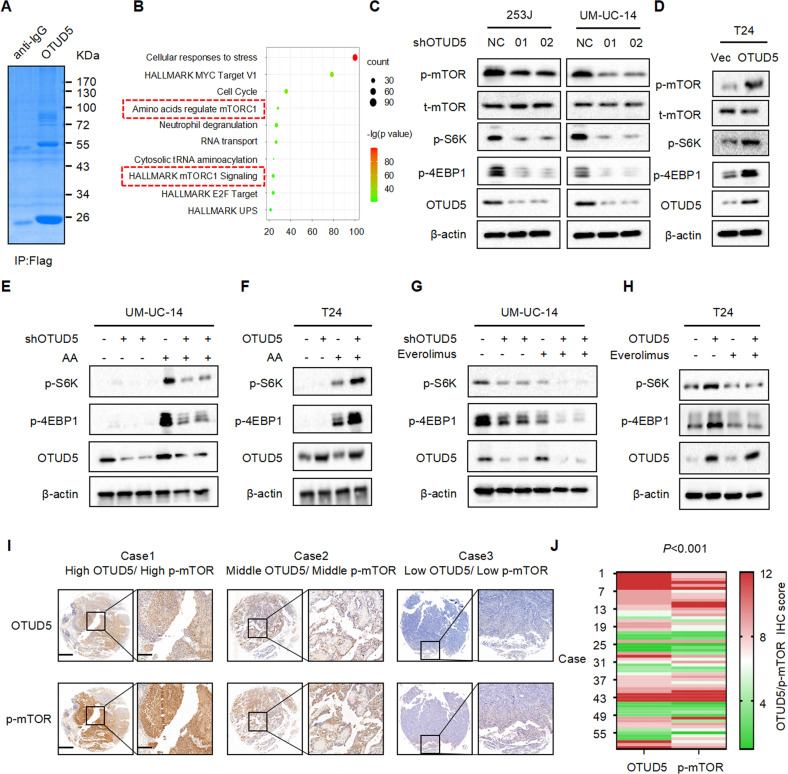
OTUD5 positively regulates the mTOR signaling pathway. **A** Flag-OTUD5 was immunoprecipitated (IP) from 293 T cells transfected with Flag-OTUD5 plasmid and the lysates were subjected to SDS-PAGE and Coomassie Blue staining. **B** Enrichment analysis of mass spectrometry results showed that OTUD5 is involved in the mTOR signaling pathway. **C** IB analysis of whole-cell lysates (WCL) derived from 253 J and UM-UC-14 cells stably expressing shOTUD5 and shNC. shNC was used as the negative control. p-mTOR, t-mTOR, p-S6K, p-4EBP1 and OTUD5 were detected. β-actin was used as the loading control. **D** IB analysis of WCL derived from T24 cells stably overexpressing OTUD5 and vector. Vector was used as the negative control. **E**, **F** UM-UC-14 shNC/shOTUD5 cells (**E**) and T24 Vec/oeOTUD5 cells (**F**) were subjected to amino acid starvation for 6 hours and resupplementation for 1 hour. p-S6K, p-4EBP1 and OTUD5 were detected. **G**, **H** UM-UC-14 shNC/shOTUD5 cells (**G**) and T24 Vec/oeOTUD5 cells (**H**) were treated with or without everolimus. **I** Representative images of OTUD5 and p-mTOR IHC staining in patient-derived bladder cancer tissues. Scale bars, left 500 μm, right 100 μm. **J**. Correlation analysis of OTUD5 and p-mTOR expression in patient-derived bladder cancer tissues. All data are presented as mean ± SD of 3 independent experiments.

Our previous results have shown that OTUD5 can promote the proliferation of bladder cancer cells. However, the underlying mechanism is still unclear. In order to explore how OTUD5 affects tumor progression, we used mass spectrometry to detect the proteins that OTUD5 may bind (Fig. 3A). Through enrichment analysis of the mass spectrometry results, it was found that OTUD5 and mTOR signaling pathway were closely related (Fig. 3B). Next, we used Western blot to verify the relationship between OTUD5 and the mTOR pathway. In 253 J and UM-UC-14 cells, compared with the control group, the expression levels of key mTOR pathway components such as p-mTOR, p-S6K and p-4EBP were reduced in the OTUD5 knockdown group (Fig. 3C). In T24 cells, compared with the control group, the expression levels of these mTOR pathway components were all increased in the OTUD5 overexpression group (Fig. 3D). Meanwhile, we performed amino acid starvation treatment in UM-UC-14 cells for 6 h, and then resumed amino acid culture for 1 h. Through Western blot detection, we found that compared with the control group, the increase in p-S6K and p-4EBP1 expression levels was less apparent in the OTUD5 knockdown groups. This suggested that knocking down OTUD5 can weaken the recovery of mTOR signaling pathway (Fig. 3E). Conversely, when amino acid was readded to the amino acid-starved T24 cells, the increase in p-S6K and p-4EBP1 expression levels was more apparent in the OTUD5 overexpression group compared with the control group. This suggested that overexpression of OTUD5 can enhance the recovery of mTOR signaling pathway (Fig. 3F). Thus, these results indicate that OTUD5 plays an important role in the regulation of mTOR signaling. Everolimus is an mTOR inhibitor and several studies have confirmed that everolimus negatively regulates mTOR signaling in other diseases. However, it has been rarely studied in bladder cancer. To confirm everolimus is regulating this intended target in bladder cancer, we examined p-S6K and p-4EBP1 levels in the bladder cancer cell lines after everolimus treatment. In UM-UC-14 cells, everolimus group showed inhibited mTOR signaling and this inhibition became even more pronounced when there was a simultaneous OTUD5 knockdown (Fig. 3G). In T24 cells, everolimus group also showed the inhibition of mTOR signaling, and this inhibition could reverse the mTOR signaling activation caused by OTUD5 overexpression (Fig. 3H). Thus, these results indicate that everolimus could negatively regulate mTOR signaling in bladder cancer cells. Moreover, the various inhibition effects in bladder cancer cells with different levels of OTUD5 expression may provide new insights into the treatment of bladder cancer in the future. Next, we investigated the relationship between p-mTOR and OTUD5 in bladder cancer tissues using immunohistochemistry. We found that p-mTOR was highly expressed in tissues with high OTUD5 expression; and p-mTOR expressed at low levels in tissues with low OTUD5 expression (Fig. 3I, J). These results indicate that the protein levels of OTUD5 and p-mTOR are positively correlated in bladder cancer tissues.

### OTUD5 regulates the protein stability of sestrin2, a feedback inhibitor of mTOR

**Fig. 4:**
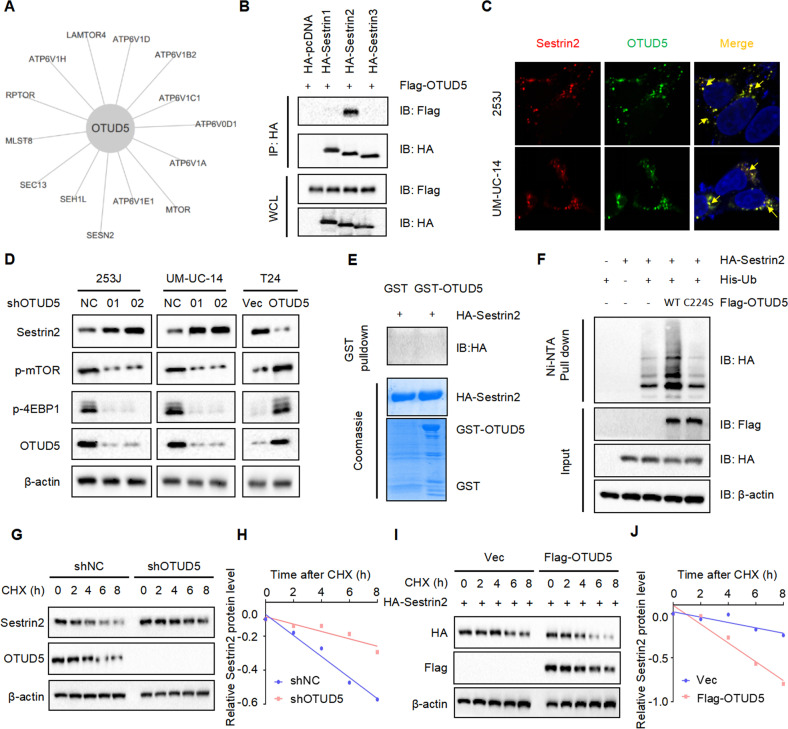
OTUD5 regulates the protein stability of sestrin2, a feedback inhibitor of mTOR. **A** The interaction diagram between OTUD5 and specific proteins in the mTOR signaling pathway was obtained by mass spectrometry analysis. **B** Confocal immunofluorescence microscopic analysis of OTUD5 and sestrin2 in 253 J and UM-UC-14 cells. Scale bars represent 10 μm. **C** IB analysis of WCL and anti-HA immunoprecipitates (IPs) derived from 293 T cells transfected with Flag-OTUD5 and HA-tagged sestrin1-3. pcDNA3.1 was used as the control. **D** IB analysis of WCL derived from 253 J and UM-UC-14 cells stably expressing shOTUD5 and T24 cells stably overexpressing OTUD5. p-mTOR, p-4EBP1 and sestrin2 were detected, β-actin was used as the loading control. **E** GST pull-down assay revealed no direct interaction between sestrin2 and OTUD5. The upper panel presents the result of IB using the antibody against HA, and the lower panels show Coomassie blue staining of the purified proteins. **F** IB analysis of WCL and Ni-NTA pull-down products derived from 293 T cells transfected with Flag-OTUD5 WT, Flag-OTUD5 C224S, HA-sestrin2 and His-Ub. 20 μM MG132 was added 6 hours before harvesting the cells. **G** OTUD5 knockdown cells (shOTUD5) as well as parental UM-UC-14 cells (shNC) were treated with 100 μg/ml cycloheximide (CHX) for the indicated time period before harvesting. Equal amounts of WCL were immunoblotted with the indicated antibodies. **H** Quantification of the band intensities in **G**. Sestrin2 levels were normalized to the corresponding β-actin levels, then normalized to the t = 0 h sestrin2 level. **I** IB analysis of WCL derived from 293 T cells transfected with HA-sestrin2, Flag-EV and Flag-OTUD5. Cells were treated with 100 μg/ml CHX for the indicated time period before harvesting. EV, empty vector. **J** Quantification of the band intensities in **I**. Sestrin2 levels were normalized to the corresponding β-actin levels, then normalized to the t = 0 h sestrin2 level. All data are presented as mean ± SD of 3 independent experiments.

Given that OTUD5 level positively correlates with p-mTOR level, we next explored the reason behind this correlation. By analyzing mass spectrometry results, we found that sestrin2 may be a potential target molecule of OTUD5 in the mTOR signaling pathway (Fig. 4A). As a feedback inhibitor in mTOR signaling pathway, sestrin2 can inhibit the function of mTOR [18, 19]. Then we found a colocalization of OTUD5 and sestrin2 by immunofluorescence (Fig. 4B) and detected a binding between OTUD5 and sestrin2 by immunoprecipitation (Fig. 4C). These results indicate that OTUD5 may bind to sestrin2 and function through deubiquitinating sestrin2. Surprisingly, when we detected the expression of sestrin2 in cell lines with stable OTUD5 knockdown and overexpression, it turned out that the expression level of sestrin2 was increased when OTUD5 was knocked down, while the expression level of sestrin2 was decreased when OTUD5 was overexpressed (Fig. 4D). These results were contradictory to our expectation that OTUD5 can stabilize sestrin2 by deubiquitination. Therefore, we speculated an indirect interaction between OTUD5 and sestrin2 instead of the direct deubiquitination. To test our speculation, we constructed the GST-OTUD5 protein and ran the GST pull down experiment. The result showed that there was no direct binding between OTUD5 and sestrin2 (Fig. 4E). Next, we investigated the possibility that OTUD5 could deubiquitinate sestrin2 through its deubiquitinating activity. Thus, we produced the wild type OTUD5 (OTUD5/WT) and the catalytically inactive OTUD5 mutant (OTUD5/C224S). In vivo ubiquitination experiment showed that OTUD5/WT could increase sestrin2 ubiquitination. While OTUD5/C224S theoretically cannot play the function of deubiquitination, the ubiquitination level of sestrin2 was reduced (Fig. 4F). Considering the role of OTUD5 as a deubiquitinase, we studied how OTUD5 affected the stability of sestrin2 by using cycloheximide (CHX). The CHX analysis showed that OTUD5 knockdown prolonged the half-life of sestrin2 (Fig. 4G, H), while OTUD5 overexpression reduced the half-life of sestrin2 (Fig. 4I, J). All these results indicate that there is no direct binding between OTUD5 and sestrin2. Since OTUD5 could not directly deubiquitinate sestrin2, we speculated that OTUD5 may regulate the expression of sestrin2 through some unknown intermediate proteins.

### OTUD5 stabilizes RNF186 by deubiquitination, leading to sestrin2 degradation

**Fig. 5:**
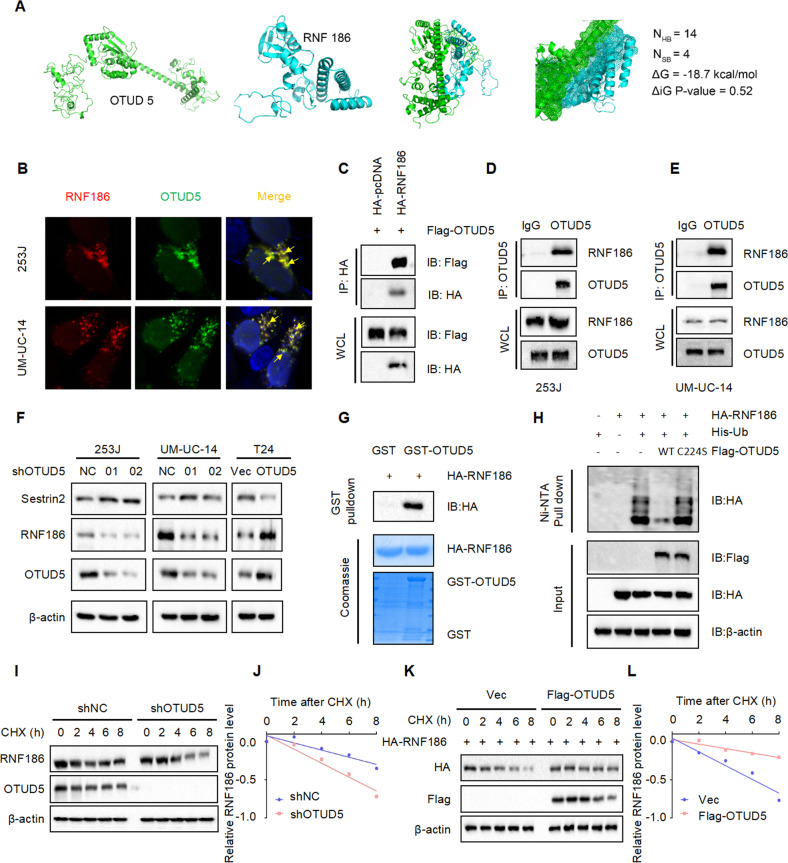
OTUD5 stabilizes RNF186 by deubiquitination, leading to sestrin2 degradation. **A** Protein binding prediction chart for OTUD5 and RNF186. **B** Confocal immunofluorescence microscopic analysis of OTUD5 and RNF186 in 253 J and UM-UC-14 cells. Scale bars represent 10 μm. **C** IB analysis of WCL and anti-HA immunoprecipitates (IPs) derived from 293 T cells transfected with Flag-OTUD5 and HA-tagged RNF186, pcDNA3.1 was used as the control. **D**, **E** OTUD5 interacts with RNF186. Co-immunoprecipitation (co-IP) of OTUD5 and RNF186 was assayed in 253 J and UM-UC-14 cells. Immunoprecipitation (IP) was performed using the antibody against OTUD5, and the endogenous interaction between OTUD5 and RNF186 was determined by Western blotting using the antibody against RNF186. **F** IB analysis of WCL derived from 253 J and UM-UC-14 cells stably expressing shOTUD5 and T24 cells stably overexpressing OTUD5. RNF186 and sestrin2 were detected, β-actin was used as the loading control. **G** GST pull-down assay revealed the direct interaction between RNF186 and OTUD5. The upper panel presents the result of IB using the antibody against HA, and the lower panels show Coomassie blue staining of the purified proteins. **H** IB analysis of WCL and Ni-NTA pull-down products derived from 293 T cells transfected with Flag-OTUD5 WT, Flag-OTUD5 C224S, HA-RNF186 and His-Ub. 20 μM MG132 was added 6 h before harvesting the cells. **I** OTUD5 knockdown cells (shOTUD5) as well as parental UM-UC-14 cells (shNC) were treated with 100 μg/ml cycloheximide (CHX) for the indicated time period before harvesting. Equal amounts of WCL were immunoblotted with the indicated antibodies. **J** Quantification of the band intensities in (**I**). RNF186 levels were normalized to the corresponding β-actin levels, then normalized to the t = 0 h RNF186 level. **K** IB analysis of WCL derived from 293 T cells transfected with HA-RNF186, Flag-EV and Flag-OTUD5. Cells were treated with 100 μg/ml CHX for the indicated time period before harvesting. EV empty vector. **L** Quantification of the band intensities in **K**. RNF186 levels were normalized to the corresponding β-actin levels, then normalized to the t = 0 h RNF186 level. All data are presented as mean ± SD of 3 independent experiments.

In order to identify the interactions between OTUD5 and sestrin2, we used protein binding prediction and found that OTUD5 may bind to RNF186. The docking results showed that OTUD5 and RNF 186 had high potential and affinity to form a complex. According to the ΔG value (−18.7 kcal/mol) of OTUD5/RNF186 complex, we predicted the binding affinity between OTUD5 and RNF 186 to be less than 0.1 nM (Fig. 5A). As an E3 ubiquitin ligase, RNF186 can affect the stability of the substrate protein. Moreover, it has been reported that it could ubiquitinate sestrin2 to control nutrient sensing [20]. Therefore, we speculated that RNF186 is the key molecule connecting OTUD5 and sestrin2. Then, we explored the potential binding between OTUD5 and RNF186 by immunofluorescence assay (Fig. 5B) and immunoprecipitation assay (Fig. 5C–E). Meanwhile, we detected the expression of RNF186 in stable OTUD5 knockdown and overexpression cell lines. The results showed the RNF186 expression decreased when OTUD5 was knocked down and increased when OTUD5 was overexpressed (Fig. 5F). This indicate that OTUD5 may directly deubiquitinate RNF186. Thus, the GST pull down experiment was performed, and the result revealed the direct binding between OTUD5 and RNF186 (Fig. 5G). In addition, the in vivo ubiquitination experiment showed that OTUD5/WT could significantly deubiquitinate RNF186, while OTUD5/C224S could not (Fig. 5H). Next, we found that OTUD5 knockdown reduced the stability of RNF186 (Fig. 5I, J), while OTUD5 overexpression prolonged the half-life of RNF186 (Fig. 5K, L). Taken together, the results we have presented so far indicate that OTUD5 stabilizes RNF186 by deubiquitination, Then, through ubiquitination, RNF186 promotes the degradation of sestrin2, which is a feedback inhibitor of the mTOR signaling pathway. Therefore, OTUD5 can regulate mTOR signaling through the OTUD5-RNF186-sestrin2-mTOR axis.

### OTUD5 knockdown combined with everolimus inhibits bladder cancer growth in vitro and in vivo

**Fig. 6:**
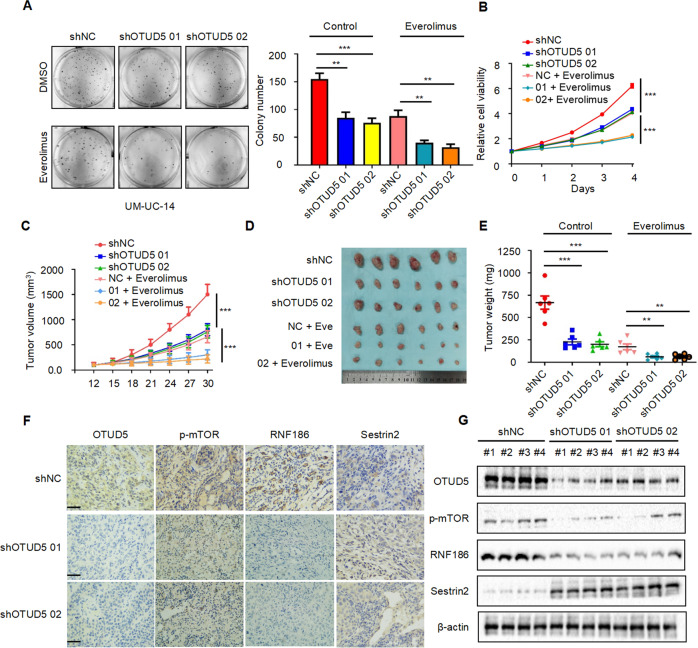
OTUD5 knockdown combined with everolimus inhibits bladder cancer growth in vitro and in vivo. **A** Colony formation assays and quantification results of UM-UC-14/WT and UM-UC-14/shOTUD5 cells treated with or without everolimus (10 nM). **P < 0.01, ***P < 0.001. **B** The growth curves of UM-UC-14/WT and UM-UC-14/shOTUD5 cells treated with or without everolimus (10 nM). ***P < 0.001. **C** The growth curves of xenografts in different treatment groups. Tumor volumes were measured every three days from day 12 to day 30. ***P < 0.001. **D** Illustration of tumors excised from male nude mice in each treatment group. **E** After 30 days, the nude mice were sacrificed, and UM-UC-14 cell xenografts were weighed. **P < 0.01, ***P < 0.001. **F** The expressions of OTUD5, p-mTOR, RNF186 and sestrin2 in the xenograft tumors were measured by immunohistochemistry. (Scale bar, 20 µm). **G** The expressions of OTUD5, p-mTOR, RNF186 and sestrin2 in the xenograft tumors were measured by Western blot. β-actin was used as the loading control. *n* = 6 mice per treatment group, data are presented as mean ± SD.

First, we investigated the in vitro inhibitory effect of OTUD5 knockdown combined with everolimus on the proliferation of bladder cancer cells. Through the plate cloning experiment (Fig. 6A) and MTT experiment (Fig. 6B), we found that compared with the NC group, OTUD5 knockdown could inhibit the proliferation of UM-UC-14 cells. Interestingly, the combination of OTUD5 knockdown and everolimus showed a more significant inhibitory effect on bladder cancer cell proliferation than the everolimus treatment alone. Then, we evaluated the in vivo inhibitory effect in a mouse model which was established by transplanting UM-UC-14 WT/shOTUD5 cells. The results showed that OTUD5 knockdown can inhibit xenograft growth, and OTUD5 knockdown combined with everolimus showed a more significant growth inhibition of the xenografts (Fig. 6C). Consistent with the tumor volume data (Fig. 6D), the same trend was observed in the tumor weight data (Fig. 6E). Furthermore, we examined the tumor tissues by Western blot (Fig. 6F) and immunohistochemistry (Fig. 6G). The results showed that compared with the control group, the expression of p-mTOR and RNF186 were downregulated while the expression of sestrin2 was upregulated in the OTUD5 knockdown group. These results are consistent with the proposed OTUD5-RNF186-sestrin2-mTOR axis and suggest OTUD5’s role in the regulation of the mTOR signaling pathway.

## Discussion

In this study, we first discovered that *OTUD5* as an oncogene promotes the progression of bladder cancer. Compared with that in normal bladder epithelial cells, OTUD5 mRNA and protein showed a trend of overexpression in bladder cancer cell lines. In addition, Western blot and immunohistochemistry found overexpressed OTUD5 in cancer tissues obtained from bladder cancer patients. Furthermore, we demonstrated that knocking down or overexpressing the *OTUD5* gene could respectively decrease or increase the growth of bladder cancer cells in clone formation and MTT experiments, indicating the cancer-promoting role of OTUD5 in bladder cancer, and suggesting a potential target for early diagnosis and treatment of bladder cancer.

Consistent with the recent published data that OTUD5 can positively regulate mTOR signaling [21], we found that OTUD5 might regulate the mTOR signaling pathway through enrichment analysis of mass spectrometry results and further confirmed the OTUD5-induced mTOR signaling activation in bladder cancer, unveiling the mechanism underlying the cancer-promoting role of OTUD5.

Since the abnormal mTOR signaling pathway is related to many diseases, especially cancer [22]. The regulation of mTOR signaling has attracted wide attention in developing treatment for these diseases. For example, everolimus as a derivative of rapamycin can significantly inhibit the activity of mTOR. It has been reported that everolimus inhibits tumor cell proliferation and induces apoptosis and autophagy [23, 24]. In addition, everolimus inhibits the malignant progression of various tumors, including breast cancer [25], ovarian cancer [26], colorectal cancer [27], pancreatic cancer [28], bladder cancer [29] and renal cell carcinoma [30]. In this study, we examined the inhibitory effect of everolimus in bladder cancer. Interestingly, our in vitro and in vivo data suggested that everolimus treatment could inhibit mTOR signaling and this inhibition becomes more significant when there is a simultaneous OTUD5 knockdown. This phenomenon indicates that OTUD5 expression levels may be used to predict everolimus sensitivity when treating bladder cancer patients in the future. Though the everolimus treatment with a simultaneous OTUD5 knockdown seems to be an ideal strategy, there is currently no specific inhibitor for OTUD5, and future discoveries of OTUD5 inhibitors may lead to some more effective bladder cancer treatments.

Further mechanistic studies of the OTUD5-induced cancer-promoting effects revealed that OTUD5 regulates mTOR signaling through the OTUD5-RNF186-sestrin2-mTOR axis in bladder cancer. First, the mass spectrometry results indicated an interaction between OTUD5 and sestrin2 which was further confirmed by IF and IP. It has been reported that sestrin2 as an inhibitor of mTOR can negatively regulate mTOR signaling [18, 31]. But interestingly, when OTUD5 was knocked down, the expression of sestrin2 increased instead. This made us wonder whether there are other proteins that play the role of a bridge between OTUD5 and sestrin2. Ring finger protein 186 (RNF186) as an E3 ubiquitin ligase can ubiquitinate the substrate protein to promote its degradation. It has been reported that RNF186 regulates ER stress-mediated apoptosis through its interaction with BNip1 [32]. Another research team has found that RNF186 can negatively regulate NF-kappaB in colorectal cancer [33]. And a recent study has shown that RNF186 can ubiquitinate and degrade sestrin2, thereby activating mTOR [20]. Our research found that OTUD5 deubiquitinates and stabilizes RNF186. Then, the stabilized RNF186 further ubiquitinates and degrades sestrin2, thereby activating mTOR signaling in bladder cancer.

**Fig. 7:**
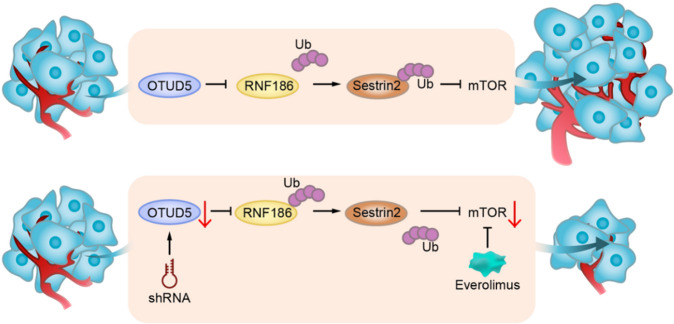
Schematic models. Upper: OTUD5 exerts its functions through the OTUD5-RNF186-sestrin2-mTOR axis to promote bladder cancer proliferation. Bottom: Downregulation of OTUD5 and everolimus show inhibitory effects on bladder cancer growth.

In summary, our research first discovered that OTUD5 plays an oncogenic role in bladder cancer. Thus, it may become a potential target for the diagnosis and treatment of bladder cancer in the future. Mechanistically, we found that OTUD5 exerts its cancer-promoting effects through the OTUD5-RNF186-sestrin2-mTOR axis. In addition, bladder cancer cells and transplanted tumors with OTUD5 knockdown are more sensitive to the mTOR inhibitor everolimus, which provides new ideas for personalized treatment of bladder cancer patients in the future (Fig. 7).

## Supplementary information

Supplementary manuscript

Supplementary figure 1

Supplementary figure 2

Supplementary figure 3

Original westeern_01

Original westeern_02

Original westeern_03

aj-checklist

Change of authorship request form

## Data Availability

All data generated and analyzed during this study are included in this published article and its additional file.
