# Supplementary material for: Deubiquitinase OTUD5 modulates mTORC1 signaling to promote bladder cancer progression
Source: Cell Death Dis. 2022 Sep 9;13(9):778. doi: 10.1038/s41419-022-05128-6 (PMC9463452; doi:10.1038/s41419-022-05128-6)
Supplement: Supplementary file 1 — Supplementary manuscript [file 41419_2022_5128_MOESM1_ESM.docx]

Supplementary Figures for

**Deubiquitinase OTUD5 modulates mTOR1 signaling to promote bladder cancer** **progression**

Tao Hou^1,2,3,^*, Weichao Dan^1,2,3,^*, Tianjie Liu^1,2,3^, Bo Liu^1,2,3^, Yi Wei^1,2,3^, Chenyang Yue^4^, Taotao Que^1,2,3^, Bohan Ma^1,2,3^, Yuzeshi Lei^1,2,3^, Zixi Wang^1,2,3^, Jin Zeng^1,2,3^, Yizeng Fan^1,2,3,#^ and Lei Li^1,2,3,#^

^1^ Department of Urology, the First Affiliated Hospital of Xi'an Jiaotong University, Xi’an, Shaanxi 710061, P.R. China

^2^ Xi'an Jiaotong university, Key Laboratory of Environment and Genes Related to Diseases, Ministry of Education, Xi'an, Shaanxi, China

^3^ The First Affiliated Hospital of Xi'an Jiaotong University, Key Laboratory of Environment and Genes Related to Diseases, Ministry of Education, Xi'an, Shaanxi, China

^4^ Department of Biology, York University, Toronto, ON M3J1P3, Canada

* Contributed equally to this work.

^#^ Correspondence to: Yizeng Fan, yzfan1991@163.com; Lei Li, E-mail: [lilydr@163.com](mailto:lilydr@163.com).

**This file includes:**

Supplementary Figure 1-3


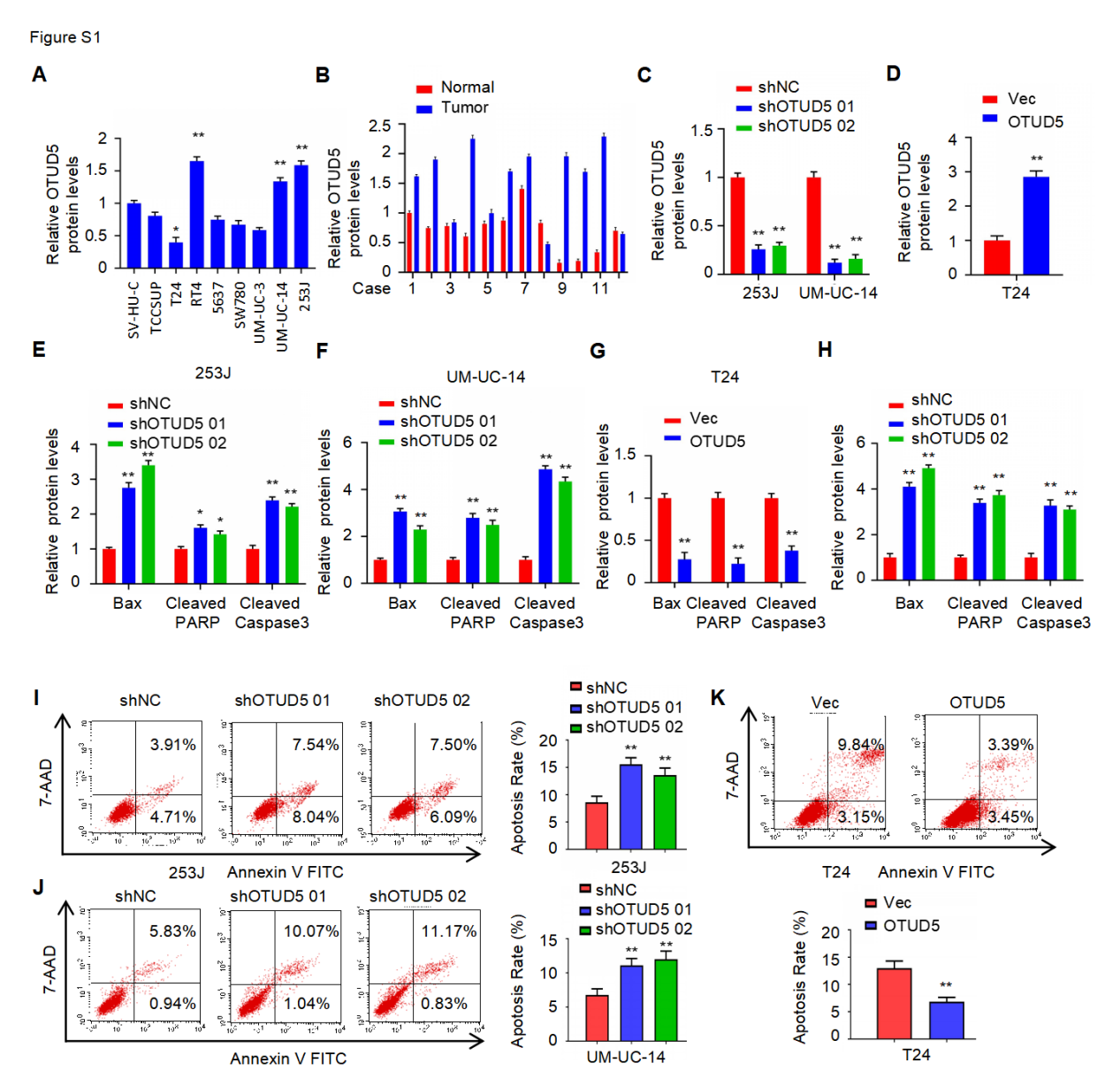


**Supplementary Figure 1. Relative protein expression quantification and normalization to actin and flow cytometry analysis. A.** Relative protein expression in figure 1C. **B.** Relative protein expression in figure 1E. **C and D.** Relative protein expression in figure 1H. **E-G.** Relative protein expression in figure 2D. **H.** Relative protein expression in figure 2I. **I-K.** Flow cytometry analysis was performed to detect apoptotic cells in 253J and UM-UC-14 cells with OTUD5 knockdown and T24 cells with OTUD5 overexpression.*P < 0.05, **P < 0.01.


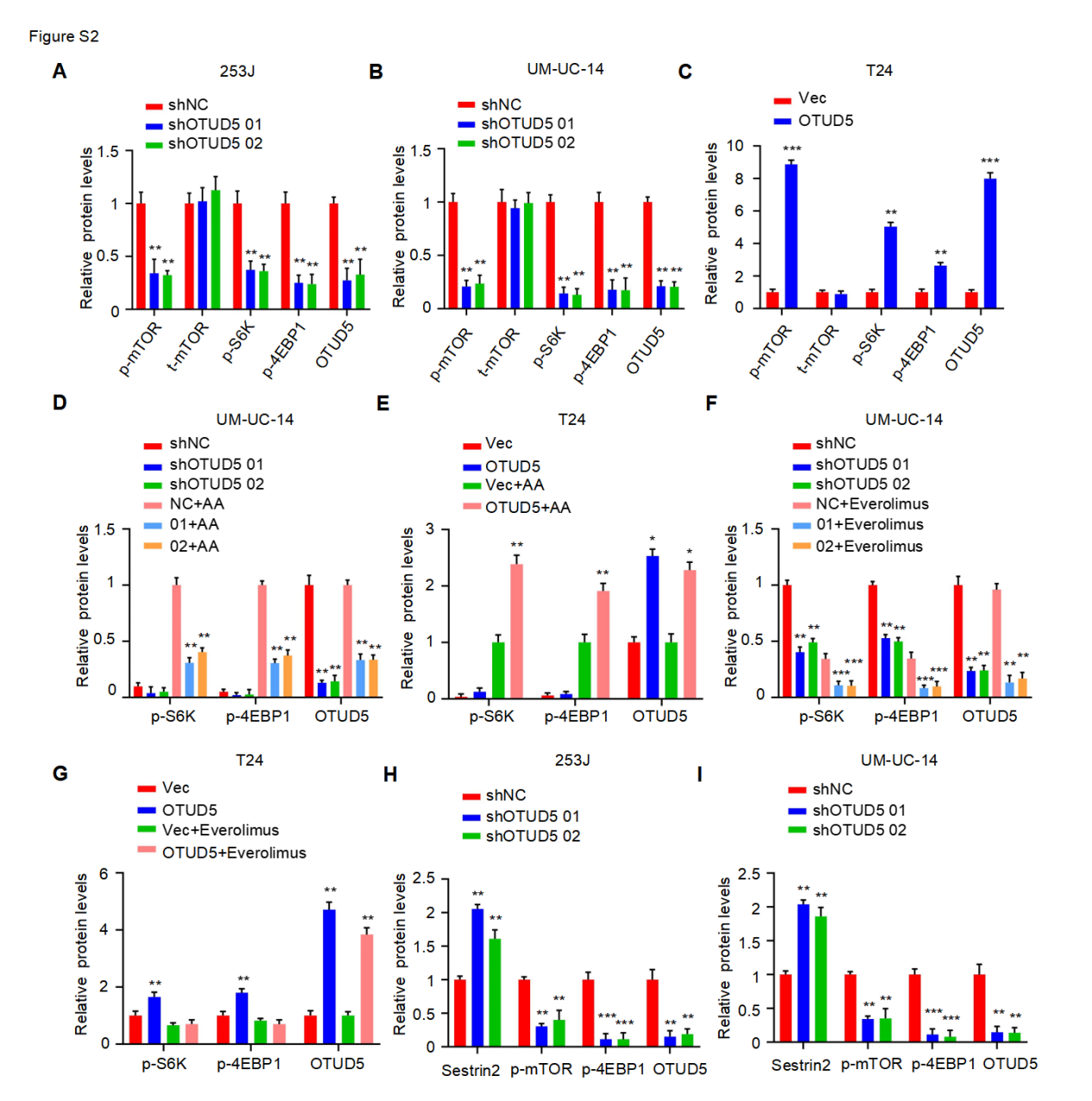


**Supplementary Figure 2. Relative protein expression quantification and normalization to actin. A-C.** Relative protein expression in figure 3C-D. **D.** Relative protein expression in figure 3E. **E.** Relative protein expression in figure 3F. **F.** Relative protein expression in figure 3G. **G.** Relative protein expression in figure 3H. **H-I.** Relative protein expression in figure 4D.


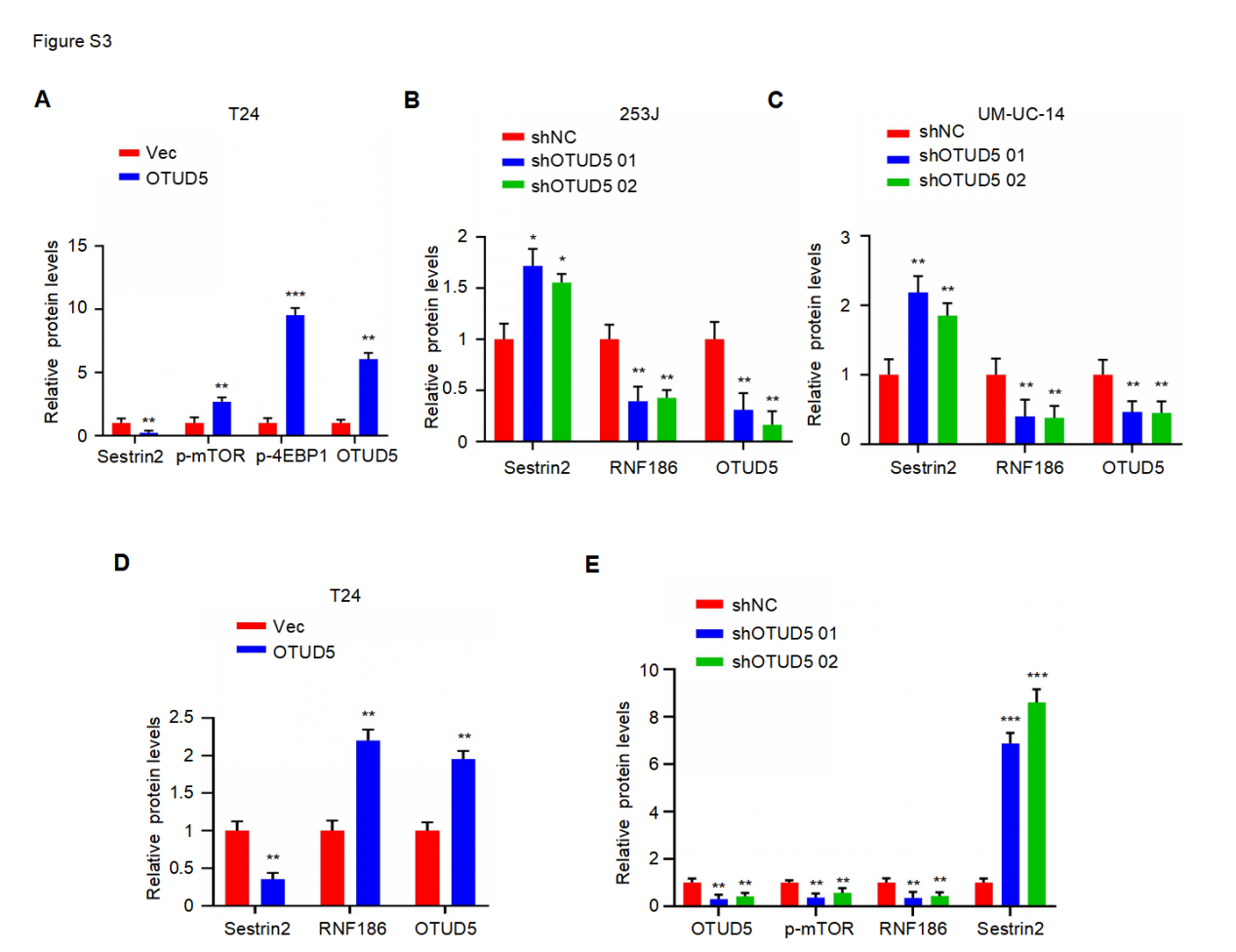


**Supplementary Figure 3. Relative protein expression quantification and normalization to actin. A.** Relative protein expression in figure 4D. **B-D.** Relative protein expression in figure 5F. **E.** Relative protein expression in figure 6G.
