# Supplementary figures and images for: Deubiquitinase OTUD5 modulates mTORC1 signaling to promote bladder cancer progression
Source: Cell Death Dis. 2022 Sep 9;13(9):778. doi: 10.1038/s41419-022-05128-6 (PMC9463452; doi:10.1038/s41419-022-05128-6)

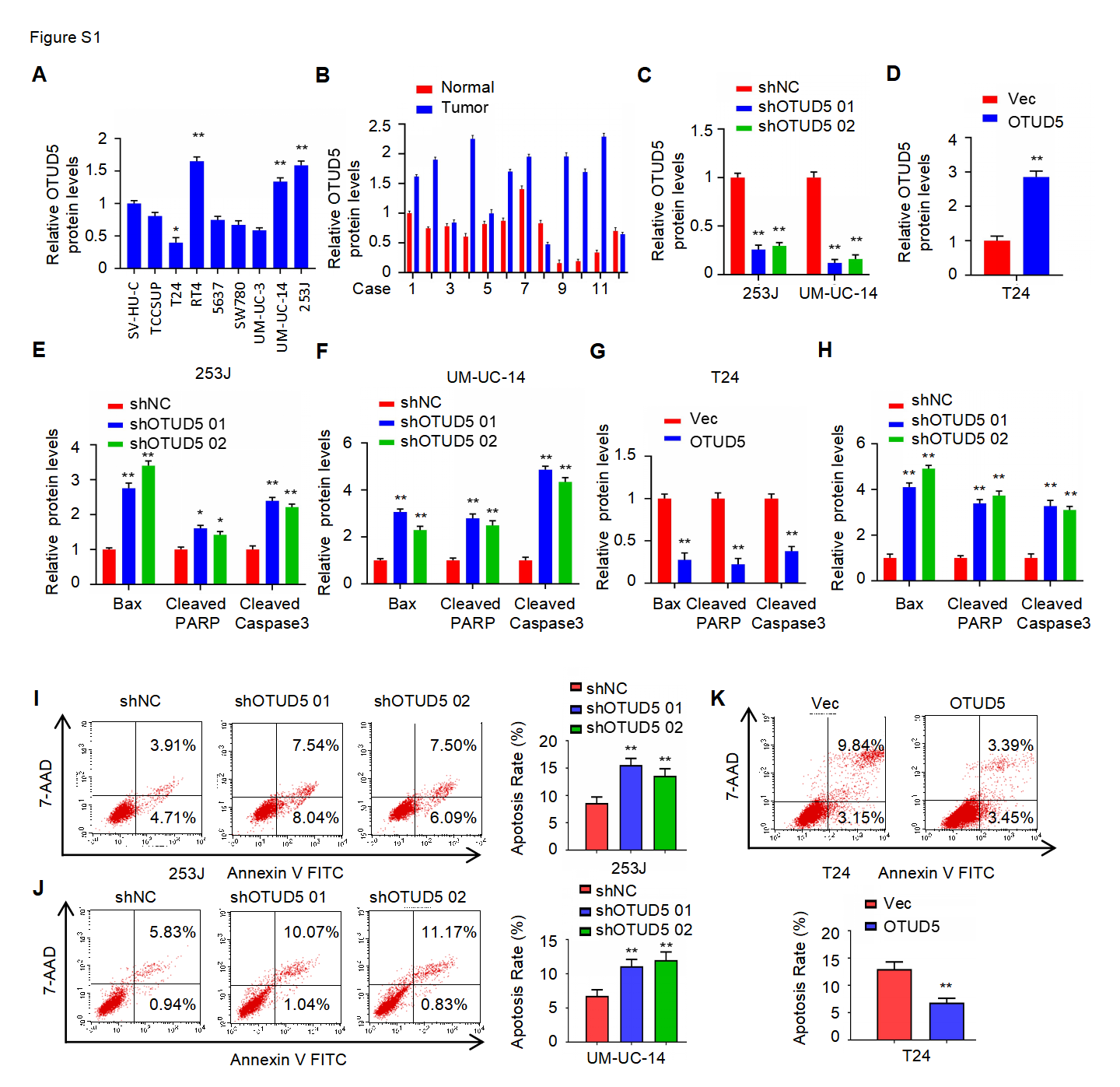

Supplement: Supplementary file 2 — Supplementary figure 1 [file 41419_2022_5128_MOESM2_ESM.tif]

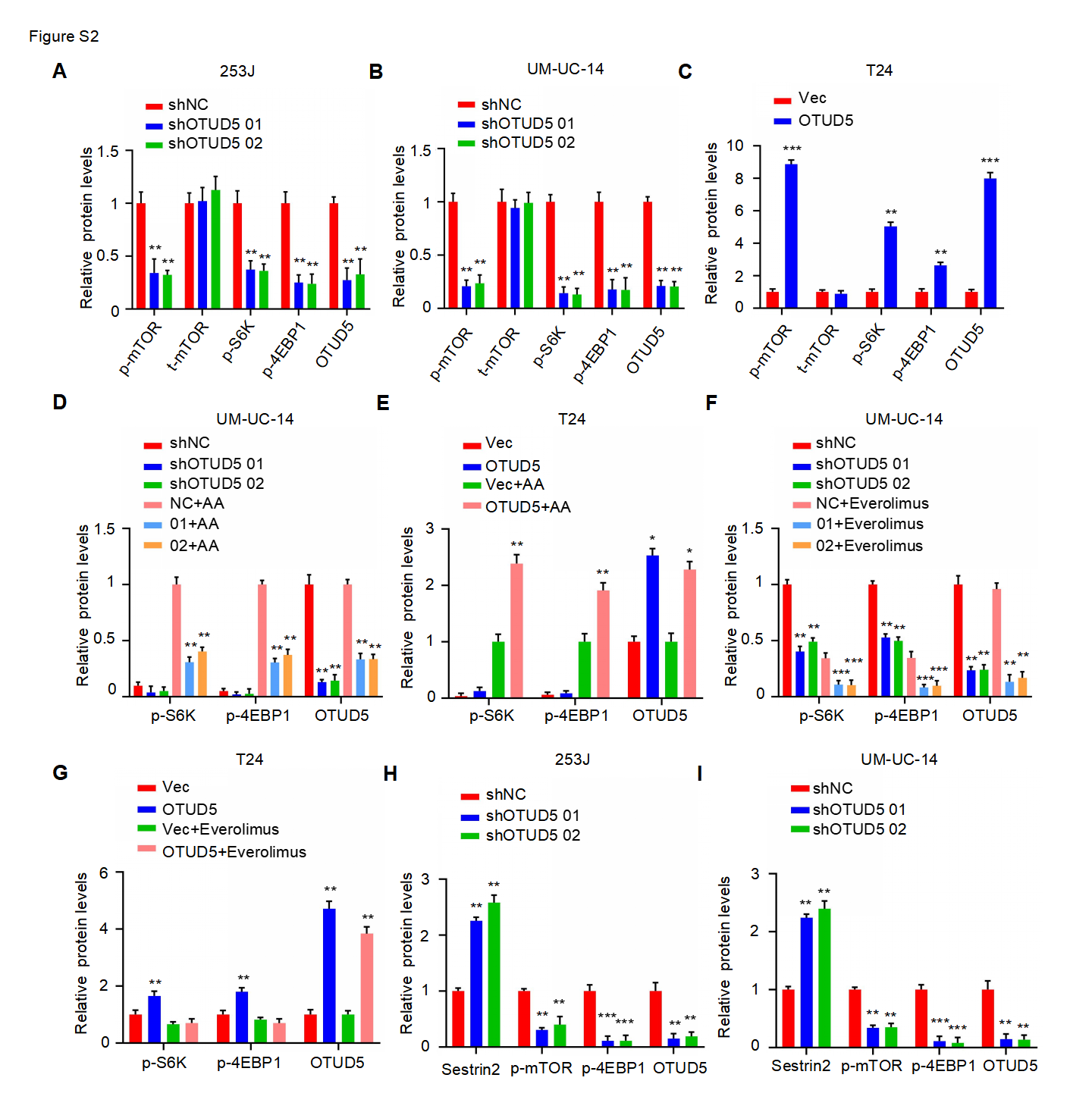

Supplement: Supplementary file 3 — Supplementary figure 2 [file 41419_2022_5128_MOESM3_ESM.tif]

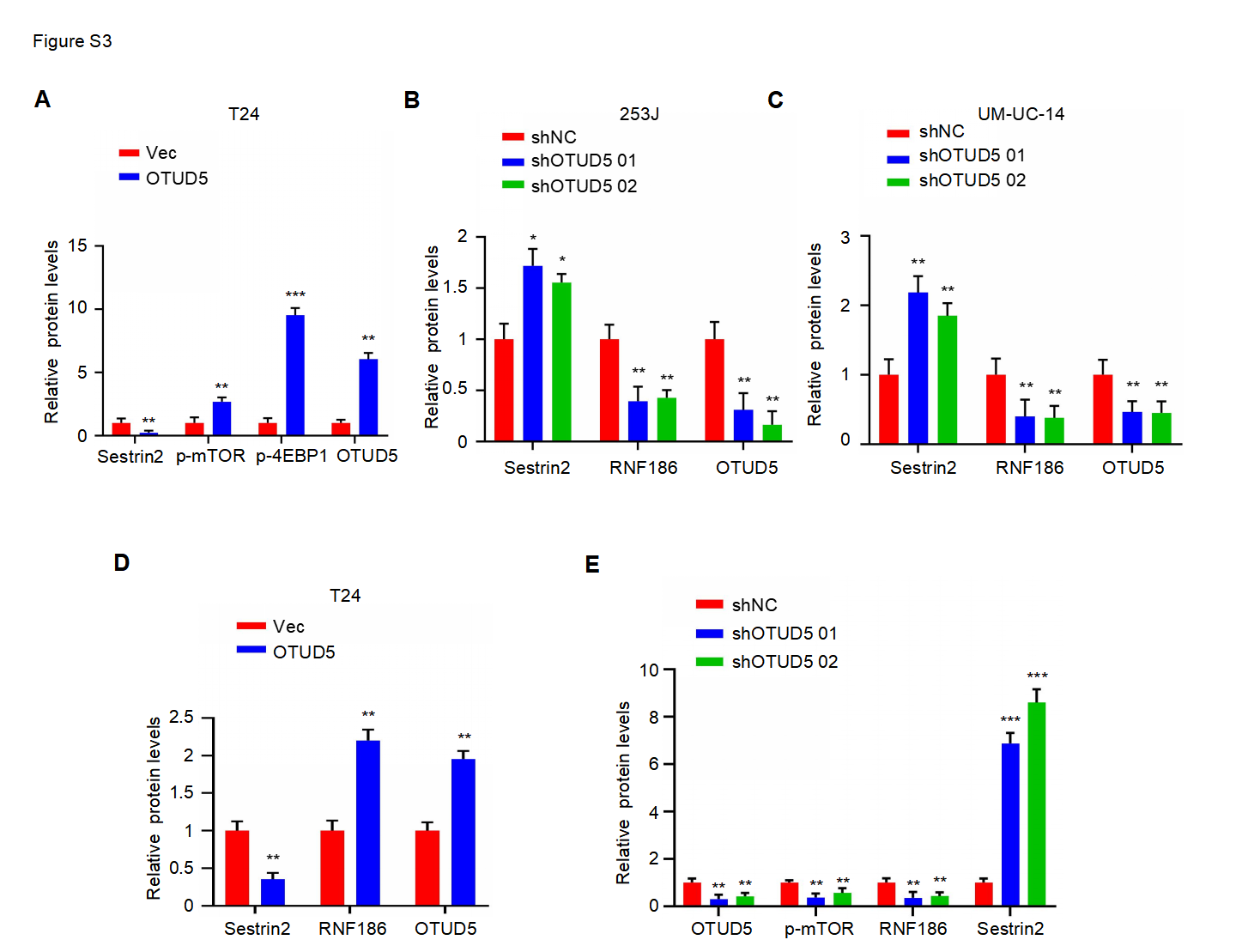

Supplement: Supplementary file 4 — Supplementary figure 3 [file 41419_2022_5128_MOESM4_ESM.tif]

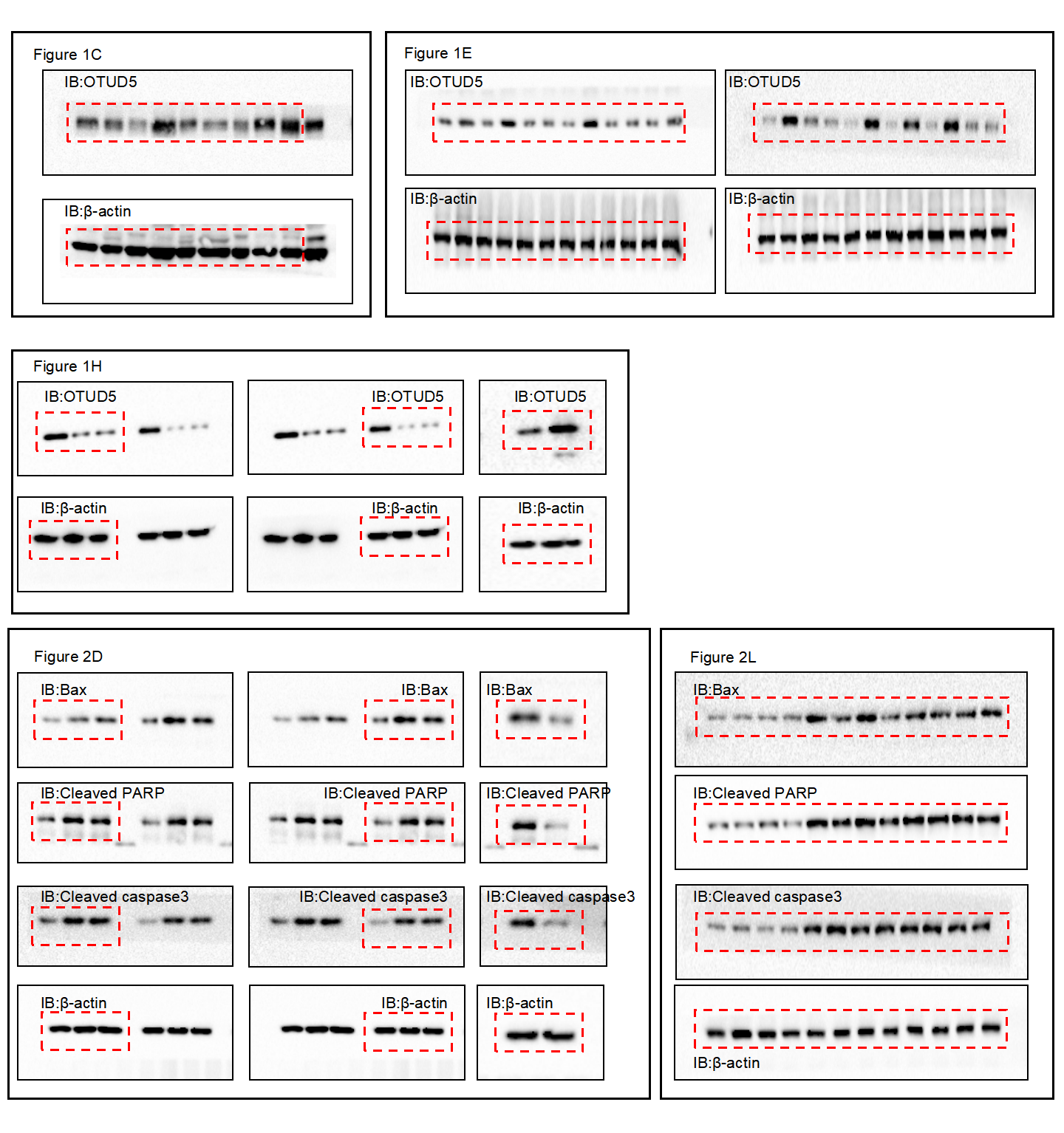

Supplement: Supplementary file 5 — Original westeern_01 [file 41419_2022_5128_MOESM5_ESM.tif]

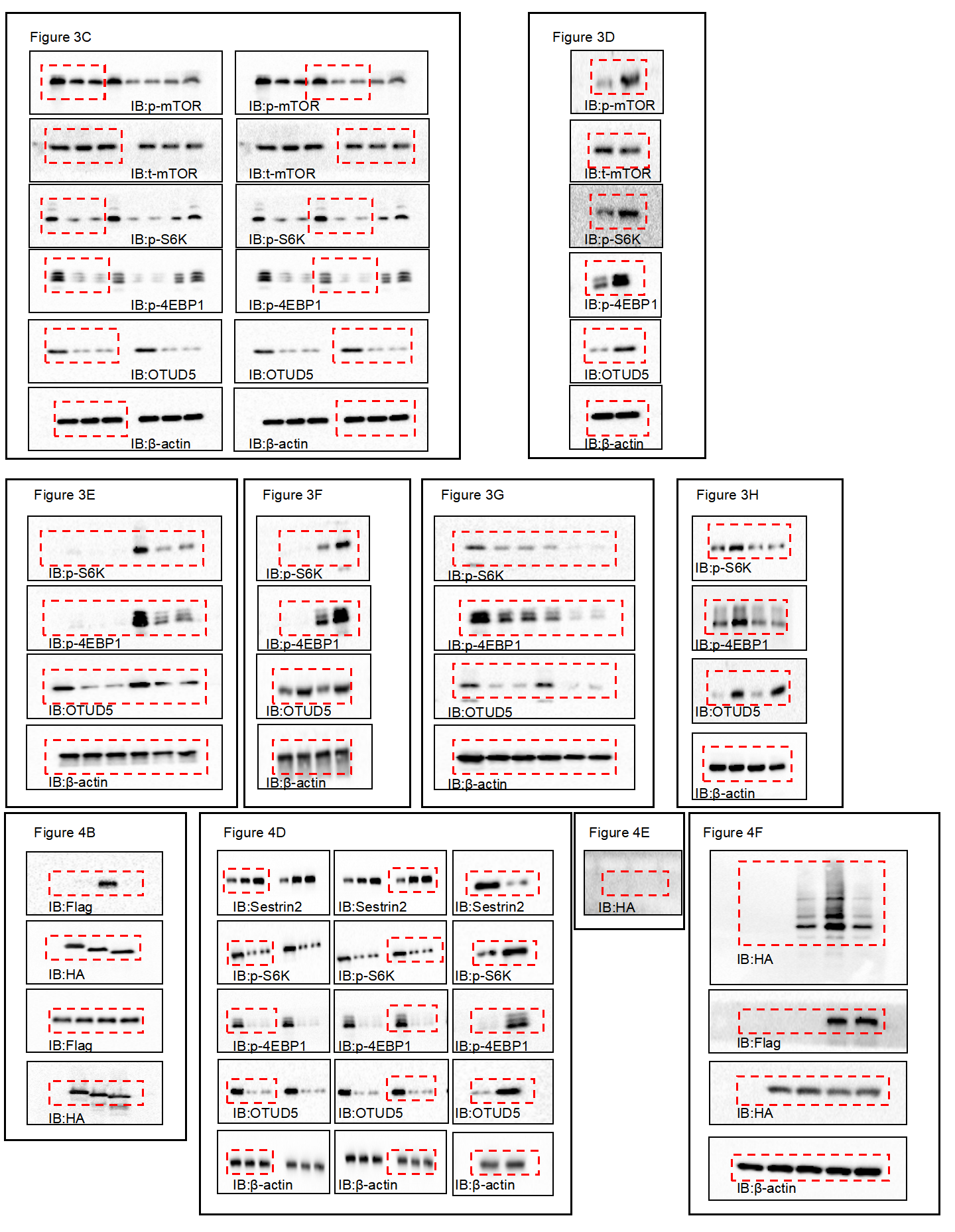

Supplement: Supplementary file 6 — Original westeern_02 [file 41419_2022_5128_MOESM6_ESM.tif]

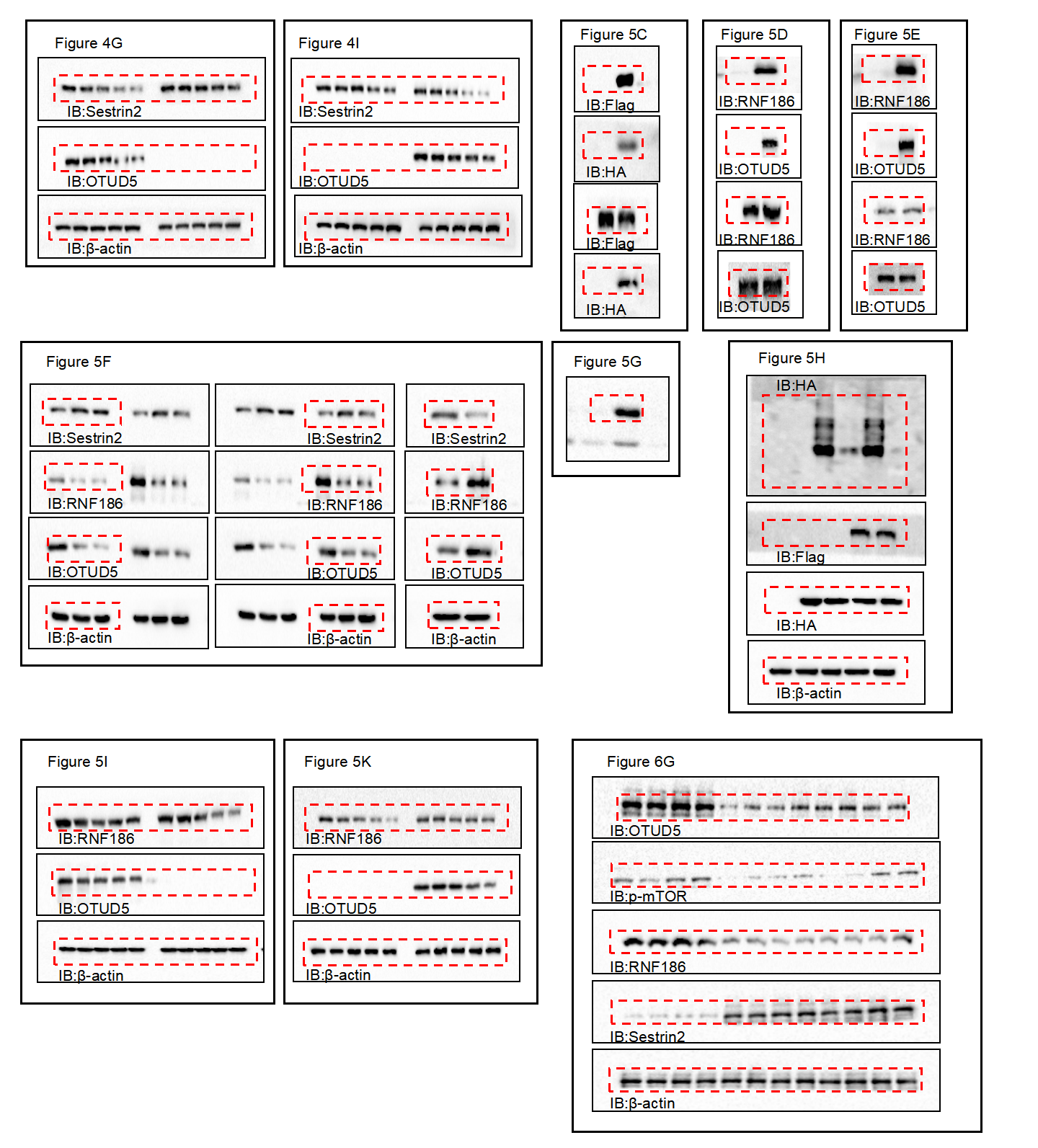

Supplement: Supplementary file 7 — Original westeern_03 [file 41419_2022_5128_MOESM7_ESM.tif]
