# Supplementary material for: Deubiquitinase OTUD5 modulates mTORC1 signaling to promote bladder cancer progression
Source: Cell Death Dis. 2022 Sep 9;13(9):778. doi: 10.1038/s41419-022-05128-6 (PMC9463452; doi:10.1038/s41419-022-05128-6)
Supplement: Supplementary file 9 — Change of authorship request form [file 41419_2022_5128_MOESM9_ESM.pdf]

SPRINGER NATURE

# Change of authorship request form - Journals (pre-acceptance)

## Important information. Please read.

- This form should be used by authors to request any change in authorship (adding/deleting authors) including changes in corresponding authors. This form should not be used for name changes. Please fully complete all sections. Use black ink and block capitals and provide each author's full name with the given name first followed by the family name.

- By signing this declaration, all authors guarantee that the order of the authors are in accordance with their scientific contribution, if applicable as different conventions apply per discipline, and that only authors have been added who made a meaningful contribution to the work.

- Please note, in author collaborations where there is formal agreement for representing the collaboration, it is sufficient for the representative or legal guarantor (usually the corresponding author) to complete and sign the Authorship Change Form on behalf of all authors, **next to the added/removed author(s)**. **(Complete Section 3, followed by Section 6.)**
  In author collaborations where there is no formal agreement for representing the collaboration and **there are more than 10 authors**, one may sign for all, provided the signer appends correspondence that attests that each of the authors have agreed to the change **and the added/removed authors sign the form**. **(Complete Section 3, followed by Section 6.)**

- Please note, we cannot investigate or mediate any authorship disputes. If you are unable to obtain agreement from all authors (including those who you wish to be removed) you must refer the matter to your institution(s) for investigation. Please inform us if you need to do this.

- If you are not able to return a fully completed form within **30 days** of the date that it was sent to the author requesting the change, we may have to withdraw your manuscript. We cannot publish manuscripts where authorship has not been agreed by all authors (including those who have been removed).

- Incomplete forms will be rejected.

- Please return/upload this form, fully completed, to the Journals Editorial Office. The Journal and/or Publisher will consider the information you have provided to decide whether to approve the proposed change in authorship. We may decide to contact your institution for more information or undertake a further investigation, if appropriate, before making a final decision.

SPRINGER NATURE

# Change of authorship request form - Journals (pre-acceptance)

**Section 1: Please provide the current title of manuscript**

**Manuscript ID no.: CDDIS-22-0635**

Title: Deubiquitinase OTUD5 modulates mTORC1 signaling to promote bladder cancer progression

**Section 2: Please provide the previous authorship, in the order shown on the manuscript before the changes were introduced. Please indicate the corresponding author by adding (CA) behind the name.**

| | First name(s) | Family name | ORCID or SCOPUS id, if available |
|---|---|---|---|
| 1st author | Tao | Hou | |
| 2nd author | Weichao | Dan | |
| 3rd author | Tianjie | Liu | |
| 4th author | Bo | Liu | |
| 5th author | Yi | Wei | |
| 6th author | Chenyang | Yue | |
| 7th author | Taotao | Que | |
| 8th author | Yuzeshi | Lei | |
| 9th author | Zixi | Wang | |
| 10th author | Jin | Zeng | |

Please use an additional sheet if there are more than 10 authors.

| | First name(s) | Family name | ORCID or SCOPUS id, if available |
|---|---|---|---|
| 11st author | Yizeng | Fan | |
| 12nd author | Lei | Li (CA) | 0000-0003-4928-8722 |
| 13rd author | | | |

---
SPRINGER NATURE Springer Nature is one of the world's leading global research, educational and professional publishers, created in May 2015 through the combination of Nature Publishing Group, Palgrave Macmillan, Macmillan Education and Springer Science+Business Media.
---

SPRINGER NATURE

# Change of authorship request form - Journals (pre-acceptance)

**Section 3: Please provide a justification for change. Please use this section to explain your reasons for changing the authorship of your manuscript, e.g. what necessitated the change in authorship? Please refer to the (journal) policy pages for more information about authorship. Please explain why omitted authors were not originally included and/or why authors were removed on the submitted manuscript.**

First, We added the new author Bohan Ma in the authorship list , for that Bohan Ma gave helpful suggestion in protein-protein docking
Second, We changed e-mail address of Yuzeshi Lei to 18302916920@163.com and e-mail address of Yizeng Fan to yzfan1991@163.com.
In addition, we regarded Yizeng Fan as the co- corresponding author. because Yizeng Fan offered helpful guidance in the revision stage.

**Section 4: Proposed new authorship. Please provide your new authorship list in the order you would like it to appear on the manuscript. Please indicate the corresponding author by adding (CA) behind the name. <u>If the Corresponding Author has changed, please indicate the reason under section 3</u>.**

| | First name(s) | Family name (this name will appear in full on the final publication and will be searchable in various abstract and indexing databases) | Affiliated institute | E-mail address |
|---|---|---|---|---|
| 1st author | Tao | Hou | the First Affiliated Hospital of Xi'an Jiaotong University | houtao1994@126.com |
| 2nd author | Weichao | Dan | the First Affiliated Hospital of Xi'an Jiaotong University | danwc1997@outlook.com |
| 3rd author | Tianjie | Liu | the First Affiliated Hospital of Xi'an Jiaotong University | liutj2016@163.com |
| 4th author | Bo | Liu | the First Affiliated Hospital of Xi'an Jiaotong University | 939305754@qq.com |
| 5th author | Yi | Wei | the First Affiliated Hospital of Xi'an Jiaotong University | wairky323@stu.xjtu.edu.cn |
| 6th author | Chenyang | Yue | York University | yue1993@my.yorku.ca |
| 7th author | Taotao | Que | the First Affiliated Hospital of Xi'an Jiaotong University | 529523780@qq.com |
| 8th author | Bohan | Ma | the First Affiliated Hospital of Xi'an Jiaotong University | bohanma@xjtu.edu.cn |
| 9th author | Yuzeshi | Lei | the First Affiliated Hospital of Xi'an Jiaotong University | 18302916920@163.com |
| 10th author | Zixi | Wang | the First Affiliated Hospital of Xi'an Jiaotong University | 948947142@qq.com |

Please use an additional sheet if there are more than 10 authors.

SPRINGER NATURE

# Change of authorship request form - Journals (pre-acceptance)

| | First name(s) | Family name (this name will appear in full on the final publication and will be searchable in various abstract and indexing databases) | Affiliated institute | E-mail address |
|---|---|---|---|---|
| 11th author | Jin | Zeng | the First Affiliated Hospital of Xi'an Jiaotong University | zengjin1984@gmail.com |
| 12th author | Yizeng | Fan (CA) | the First Affiliated Hospital of Xi'an Jiaotong University | yzfan1991@163.com |
| 13th author | Lei | Li (CA) | the First Affiliated Hospital of Xi'an Jiaotong University | lilydr@163.com |

---
SPRINGER NATURE Springer Nature is one of the world's leading global research, educational and professional publishers, created in May 2015 through the combination of Nature Publishing Group, Palgrave Macmillan, Macmillan Education and Springer Science+Business Media.
---

# Change of authorship request form - Journals (pre-acceptance)

**Section 5: Author contribution, Acknowledgement and Disclosures. Please use this section to provide a new disclosure statement and, if appropriate, acknowledge any contributors who have been removed as authors and ensure you state what contribution any new authors made (if applicable per the journal or book (series) policy).** Please ensure these are updated in your manuscript - after approval of the change(s) - as our production department will not transfer the information in this form to your manuscript.

**New acknowledgements:**

Not applicable

**New Disclosures (financial and non-financial interests, funding):**

Funding:
This study was supported by National Natural Science Foundation of China (NO.81925028 and NO.82002694).

Conflict of interest:

The authors declare that they have no conflict of interest.

**New Author Contributions statement (if applicable per the journal policy):**

T.H. and W.C.D. contributed equally to this work. J.Z., Y.Z.F. and L.L. designed and supervised all the experiments and contributed to manuscript preparation.

T.H., W.C.D., T.J.L., B.L., Y.W., T.T.Q., B.H.M., Y.Z.S.L. and Z.X.W. performed the experiments, analysed the data and contributed to the manuscript preparation.

T.H. and W.C.D. collected animal samples and participated in the animal experiments. T.H., C.Y.Y., Y.Z.F. and L.L. wrote the manuscript.

State 'Not applicable' if there are no new authors.

---
SPRINGER NATURE Springer Nature is one of the world's leading global research, educational and professional publishers, created in May 2015 through the combination of Nature Publishing Group, Palgrave Macmillan, Macmillan Education and Springer Science+Business Media.
---

SPRINGER NATURE

# Change of authorship request form - Journals (pre-acceptance)

**Section 6: Declaration of agreement. *All* authors, unchanged, new and removed *must* sign this declaration.**
(NB: Please print the form, (docu)-sign and return/upload a scanned copy. Please note that signatures that have been inserted as an image file are acceptable as long as it is handwritten. Typed names in the signature box are unacceptable.) *** Please delete as appropriate. Delete all of the bold if you were on the original authorship list and are remaining as an author.**

| | First name | Family name | | Signature | Date |
|---|---|---|---|---|---|
| 1st author | Tao | Hou | I agree to the proposed new authorship shown in section 4 **/and the addition/removal*of my name to the authorship list** /and the proposed change in corresponding author | Tao Hou | 2022.6.27 |
| 2nd author | Weichao | Dan | I agree to the proposed new authorship shown in section 4 **/and the addition/removal*of my name to the authorship list** /and the proposed change in corresponding author | Dan Weichao | 2022.6.27 |
| 3rd author | Tianjie | Liu | I agree to the proposed new authorship shown in section 4 **/and the addition/removal*of my name to the authorship list** /and the proposed change in corresponding author | Tianjie Liu | 2022.6.27 |
| 4th authors | Bo | Liu | I agree to the proposed new authorship shown in section 4 **/and the addition/removal*of my name to the authorship list** /and the proposed change in corresponding author | Liu Bo | 2022.6.27 |
| 5th author | Yi | Wei | I agree to the proposed new authorship shown in section 4 **/and the addition/removal*of my name to the authorship list** /and the proposed change in corresponding author | Yi Wei | 2022.6.27 |
| 6th author | Chenyang | Yue | I agree to the proposed new authorship shown in section 4 **/and the addition/removal*of my name to the authorship list** /and the proposed change in corresponding author | Chenyang Yue | 2022.6.27 |
| 7th author | Taotao | Que | I agree to the proposed new authorship shown in section 4 **/and the addition/removal*of my name to the authorship list** /and the proposed change in corresponding author | Taotao Que | 2022.6.27 |

---
SPRINGER NATURE Springer Nature is one of the world's leading global research, educational and professional publishers, created in May 2015 through the combination of Nature Publishing Group, Palgrave Macmillan, Macmillan Education and Springer Science+Business Media.
---

SPRINGER NATURE

# Change of authorship request form - Journals (pre-acceptance)

| | First name | Family name | | Signature | Date |
|---|---|---|---|---|---|
| 8th author | Bohan | Ma | I agree to the proposed new authorship shown in section 4 **/and the addition/removal*of my name to the authorship list** /and the proposed change in corresponding author | Bohan Ma | 2022-06.27 |
| 9th author | Yuzeshi | Lei | I agree to the proposed new authorship shown in section 4 **/and the addition/removal*of my name to the authorship list** /and the proposed change in corresponding author | Yuzeshi Lei | 2022.06.27 |
| 10th author | Zixi | Wang | I agree to the proposed new authorship shown in section 4 **/and the addition/removal*of my name to the authorship list** /and the proposed change in corresponding author | Zixi Wang | 2022-06-27 |

Please use an additional sheet if there are more than 10 authors.

| | First name | Family name | | Signature | Date |
|---|---|---|---|---|---|
| 11th author | Jin | Zeng | I agree to the proposed new authorship shown in section 4 **/and the addition/removal*of my name to the authorship list** /and the proposed change in corresponding author | Jin Zeng | 2022.06.27 |
| 12th author | Yizeng | Fan (CA) | I agree to the proposed new authorship shown in section 4 **/and the addition/removal*of my name to the authorship list** /and the proposed change in corresponding author | Yizeng Fan | 2022-06-27 |
| 13th author | Lei | Li (CA) | I agree to the proposed new authorship shown in section 4 **/and the addition/removal*of my name to the authorship list** /and the proposed change in corresponding author | Lei Li | 2022-06-27 |

**In case of author collaborations with formal agreement:**

---
SPRINGER NATURE Springer Nature is one of the world's leading global research, educational and professional publishers, created in May 2015 through the combination of Nature Publishing Group, Palgrave Macmillan, Macmillan Education and Springer Science+Business Media.
---

# Change of authorship request form - Journals (pre-acceptance)

| | Name of consortium/consortia | First name | Family name | | Signature | Date |
|---|---|---|---|---|---|---|
| Representative/legal guarantor | | | | I agree to the proposed new authorship shown in section 4 **/and the addition/removal*of my name to the authorship list** /and the proposed change in corresponding author | | |

**Both added/removed authors should complete the information in the first table under Section 6.**

---- End of form ----
